# A Novel Proton Pencil Beam Scanning FLASH RT Delivery Method Enables Optimal OAR Sparing and Ultra-High Dose Rate Delivery: A Comprehensive Dosimetry Study for Lung Tumors

**DOI:** 10.3390/cancers13225790

**Published:** 2021-11-18

**Authors:** Shouyi Wei, Haibo Lin, J. Isabelle Choi, Charles B. Simone, Minglei Kang

**Affiliations:** New York Proton Center, New York, NY 10035, USA; awei@nyproton.com (S.W.); hlin@nyproton.com (H.L.); ichoi@nyproton.com (J.I.C.); csimone@nyproton.com (C.B.S.II)

**Keywords:** proton therapy, pencil beam scanning, transmission beam, proton Bragg peak FLASH, ultra-high dose rate, FLASH radiotherapy, lung hypofractionation

## Abstract

**Simple Summary:**

This study attempts to answer a novel and clinically relevant question of the value of Bragg-peak-based FLASH planning for lung tumors. Most existing studies and literature are limited to using transmission proton beams at ultra-high dose rates, resulting in unnecessary irradiation exposure to normal tissues beyond the target volume. By combining a new hardware design (universal range shifter and range compensator) and an inverse planning system, the novel Bragg peak method makes the Bragg-peak-based FLASH planning possible. The treatment planning study and dosimetry comparison between single-energy proton Bragg peak beams and transmission proton beams demonstrated superior performances in OAR sparing and comparable FLASH dose rate of the Bragg peak FLASH. Beam angle optimization can further improve Bragg peak FLASH dosimetry performance while maintaining the similar 3D FLASH dose rate coverage for OARs.

**Abstract:**

Purpose: While transmission proton beams have been demonstrated to achieve ultra-high dose rate FLASH therapy delivery, they are unable to spare normal tissues distal to the target. This study aims to compare FLASH treatment planning using single energy Bragg peak proton beams versus transmission proton beams in lung tumors and to evaluate Bragg peak plan optimization, characterize plan quality, and quantify organ-at-risk (OAR) sparing. Materials and Methods: Both Bragg peak and transmission plans were optimized using an in-house platform for 10 consecutive lung patients previously treated with proton stereotactic body radiation therapy (SBRT). To bring the dose rate up to the FLASH-RT threshold, Bragg peak plans with a minimum MU/spot of 1200 and transmission plans with a minimum MU/spot of 400 were developed. Two common prescriptions, 34 Gy in 1 fraction and 54 Gy in 3 fractions, were studied with the same beam arrangement for both Bragg peak and transmission plans (*n* = 40 plans). RTOG 0915 dosimetry metrics and dose rate metrics based on different dose rate calculations, including average dose rate (ADR), dose-averaged dose rate (DADR), and dose threshold dose rate (DTDR), were investigated. We then evaluated the effect of beam angular optimization on the Bragg peak plans to explore the potential for superior OAR sparing. Results: Bragg peak plans significantly reduced doses to several OAR dose parameters, including lung V_7.4Gy_ and V_7Gy_ by 32.0% (*p* < 0.01) and 30.4% (*p* < 0.01) for 34Gy/fx plans, respectively; and by 40.8% (*p* < 0.01) and 41.2% (*p* < 0.01) for 18Gy/fx plans, respectively, compared with transmission plans. Bragg peak plans have ~3% less in DADR and ~10% differences in mean OARs in DTDR and DADR relative to transmission plans due to the larger portion of lower dose regions of Bragg peak plans. With angular optimization, optimized Bragg peak plans can further reduce the lung V_7Gy_ by 20.7% (*p* < 0.01) and V_7.4Gy_ by 19.7% (*p* < 0.01) compared with Bragg peak plans without angular optimization while achieving a similar 3D dose rate distribution. Conclusion: The single-energy Bragg peak plans achieve superior dosimetry performances in OARs to transmission plans with comparable dose rate performances for lung cancer FLASH therapy. Beam angle optimization can further improve the OAR dosimetry parameters with similar 3D FLASH dose rate coverage.

## 1. Introduction

FLASH radiation therapy (RT), characterized by an ultra-high dose rate of >40 Gy/s, has the potential to offer superior normal tissue sparing while retaining similar tumor control to conventional dose rate RT [1,2,3,4]. Recently, several pioneering pre-clinical studies have corroborated the FLASH effect using electron beams in mice models with lung [3], brain [1,5,6], and mini-pig and cat patients [7]. The first human patient treated with FLASH electron beams also achieved total and rapid tumor response with limited skin effects, validating the efficacy of FLASH RT [8]. FLASH RT using proton beams has been implemented using scattering systems, also showing promising pre-clinical treatment outcomes [9,10,11]. Pencil beam scanning (PBS), the most advanced proton beam delivery technique, is capable of achieving extraordinary dose conformity by steering narrow proton beamlets with scanning magnets. Major proton vendors have upgraded their PBS proton systems, which are capable of delivering ultra-high nozzle beam currents to reach FLASH dose rates [12,13,14,15]. Recently, the first FLASH clinical trial has been activated using a proton accelerator [16,17].

Currently, there is no consensus in PBS dose rate definition, and a variety of dose rate calculation methods have been proposed for 3D dose rate assessment for FLASH RT planning studies [18,19,20]. Unlike double scattering (DS) proton or electron treatment, the dose delivery in a PBS field runs via scanning magnets, which scan hundreds of spots to cover the entire target layer-by-layer. As a result, the dose rate quantification is more complex for PBS, since more factors need to be accounted for, such as inter-spot switch time, dose threshold, spot dose weighting effect, etc. It is currently unclear how each of these factors relates to the FLASH effect. Furthermore, in the delivery of each proton spot (i.e., beamlet), the proton bunches are modulated by pulse sequences of variable widths and intervals, giving rise to even more complicated dose rate characteristics [21]. For the ProBeam system (Varian Medical Systems, Palo Alto, CA, USA), the intra-spot proton delivery could be approximated as a continuous process [21], while for other systems, such as the Hyperscan proton system (Mevion Medical Systems, Littleton, MA, USA) [21,22], the interval between bunches may not be neglected, as it is usually relatively long.

Several previous treatment planning studies have investigated factors that affect overall plan quality for proton FLASH systems, such as dose uniformity in target and organs-at-risks (OARs) and dose rates. The first published study [18], which implemented transmission plans with 229 MeV proton beams for head and neck patients, proposed and investigated dose-averaged dose rates (DADR) achieved with current and ideal machine settings. Similarly, a later study [23] applied transmission plans using 244 MeV proton beams to lung cancer patients, which obtained superior plan quality to volumetric modulated arc therapy (VMAT) and also assessed dose rate, irradiating time, and dose rate-dose relations for the plans. As the gaps between planning and available machine delivery were not fully understood, Zou et al. [24] investigated and discussed more realistic machine-related delivery limitations that affected the achievable dose rates in PBS, including beam current, spot dwelling time, and energy switch. Another important aspect for addressing FLASH planning-related challenges is to achieve inverse planning for OAR FLASH dose rate coverage; an algorithm proposed by Gao et al. [25] may offer a promising solution to optimize DADR dose rate volume coverage in OARs. Verhaegen et al. [26] investigated transmission FLASH proton treatment plans by assuming a normal tissue protection factor of two, and their results suggested significant dose reduction in normal tissues while conforming to the dose constraints without beam optimization. Kang et al. [20] studied the intensity-modulated proton therapy (IMPT) transmission plans for hypofractionated lung cancer based on beam parameters, including the beam current and minimum spot delivery time, to fully assess the dosimetry and dose rate performances using multiple single-energy proton beams.

Based on the above studies, single-energy proton beam transmission plans achieve good target uniformity and high dose rate coverage in OARs. Furthermore, it is more feasible to use single energy to reach a higher beam current, compared to lower energies, and to avoid the long switch-time between energy layers.

Nevertheless, the disadvantages of transmission plans may not be ignored, as they will not be able to spare the normal tissue distal to the target in the beam path as they traverse the entire body. For large patients and certain target locations, beams with the highest energy may not shoot through at certain angles, causing overdose in normal tissues, adding to planning challenges, and affecting the overall plan quality. Moreover, recent studies [27,28,29] indicate non-significant differences in treatment effects between FLASH and conventional irradiation when the delivered dose is relatively low, typically close to or less than 5 Gy. The above challenges can be addressed when combining the advantages of IMPT, which places the Bragg peak within the target, and single-energy delivery, which enables the FLASH dose rate. Therefore, single-energy Bragg peak plans, offering high dose delivery, along with rapid distal dose fall-off in the Bragg peak, can completely spare normal tissues distal to the target compared to transmission plans, making it an attractive alternative for FLASH treatment planning.

In this work, we implement a novel method using Bragg peaks for FLASH treatment planning to eliminate the exit dose and better spare normal tissues for hypofractionated lung tumor treatment, and evaluate its feasibility to achieve an ultrahigh dose rate suitable for FLASH. The dosimetric characteristics and 3D dose rate distribution of single-energy Bragg peak plans and transmission plans are investigated. The study aims to quantify the dosimetry advantages of single-energy Bragg peak plans. The Bragg peak planning method has the potential to yield superior plan quality while still achieving sufficient FLASH dose rate coverage, which can be potentially applied for future lung cancer clinical trials and FLASH studies.

## 2. Methods and Materials

### 2.1. Development of Single-Energy Bragg Peak Planning

The first proof-and-concept studies using Bragg peak of a single-energy proton for FLASH RT treatment planning have recently been investigated by our group [30]. To adapt the Bragg peak of proton beams to the target distal edge, we have developed a universal range shifter combined with the beam-specific range compensators to pull each proton beamlet back at the designed depths. This allows for the elimination of exit dose and better OAR sparing. The unit of Gy includes a constant 1.1 RBE factor in this study.

The Varian ProBeam beam models were configured in our in-house platform. In a previous study, Folkerts et al. [19] adopted a 2 ms delivery time for the minimum MU/spot and 10 mm/ms scanning speed under FLASH mode for the Varian ProBeam system [19]. Thus, the current planning study is based on the above assumptions. van Marlen et al. [23] first defined spot peak dose rate (SPDR) as the max dose rate of the central axis of a spot to quantify single spot dose rate. Based on the beam current and delivery mechanism, we can quantify the dose rates using different proposed metrics, including DADR [18], ADR [19], and DTDR [20]. A 2 ms delivery time and ~140 nA beam current in the treatment room corresponds to a minimum MU/spot of 400 and ~640 Gy/s SPDR [20]. Recently, transmission efficiency of 86% from the cyclotron to the treatment room [31] was achieved for the PSI gantry 1, and if similar efficiency is assumed for the ProBeam system, then the maximum nozzle beam current is ~690 nA, equivalent to a minimum MU/spot of ~1970 assuming a 2 ms delivery time and ~2800 Gy/s SPDR in the treatment room. The SPDR in the near-Bragg peak region is typically ~40% lower compared to the plateau region for a single spot [20], especially in the presence of an air gap between the range shifter and the patient surface [20]. Therefore, using Bragg peak planning is necessary to use a high minimum MU/spot, i.e., higher nozzle current, to achieve a sufficient dose rate. This work explores the dosimetric potential by applying a different minimum MU/spot under currently realistic and achievable machine settings [19,31] for Bragg peak plans compared to transmission plans.

To achieve acceptable plan quality and ultra-high dose rates for the Bragg peak plans, we developed a novel method for spot map and dose rate optimization through inverse IMPT planning. The Bragg peak plans were first optimized by setting a small minimum MU/spot that ensures plan quality. Then, we applied a spot map optimization algorithm to merge low-weighted spots with nearby spots in the initial optimized plans. The details of the algorithm are described in [30]. The fields, now with merged spots, were optimized again with the higher minimum MU/spot constraint. Plan quality, especially target uniformity, may degrade after dose rate optimization, and in such cases, the original spot spacing can be adjusted, and the above Bragg peak planning process was iteratively optimized until an acceptable balance between plan quality and optimized dose rate was achieved.

### 2.2. FLASH Transmission and Bragg Peak Planning

**Figure 1:**
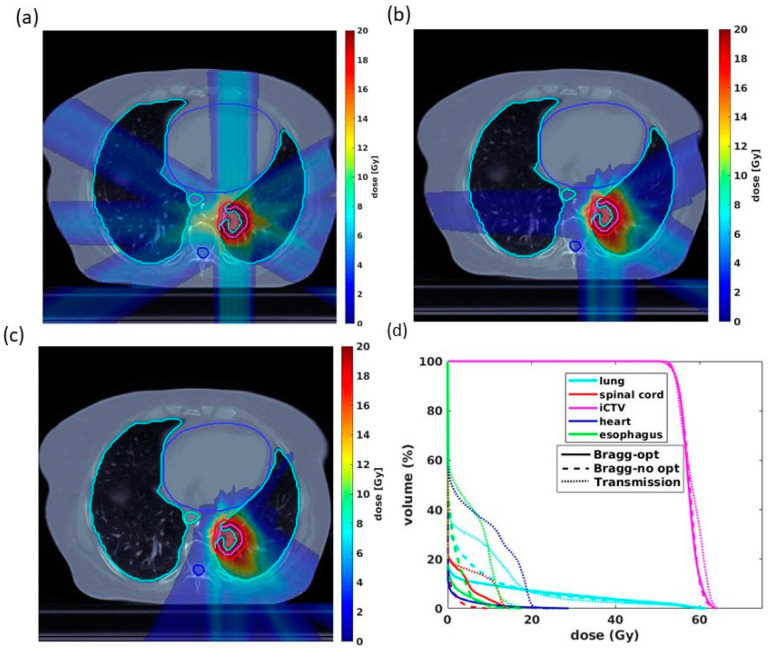
Dosimetry comparison between transmission and Bragg peak proton FLASH plans for one representative case. (**a**) Transmission plans with 4 beam angles with each angle separation. (**b**) Bragg peak plans with the same arrangement as transmission plans. (**c**) Bragg peak plans with an optimized beam angle arrangement better approximating current conventional-dose rate PBS plans used in clinical practice. (**d**) DVHs of the three plans, demonstrating differences in OARs and target dose coverage.

This study was conducted under institutional review board (IRB) approval. A cohort of 10 consecutive lung cancer patients previously treated at our institution by proton stereotactic body radiation therapy (SBRT) was replanned using transmission and Bragg peak FLASH beams for this study. The plans were developed with 34 Gy in 1 single fraction and 54 Gy in 3 fractions, two common standard-of-care dose fractionation regimens used in prior cooperative group SBRT lung cancer trials [32]. Gross tumor volume (GTV) is visible, palpable, or demonstratable through diagnostic imaging. A clinical margin encompassing potential areas of microscopic disease was added to the GTV to generate the CTV. The internal clinical target volume (iCTV) was generated on an averaged CT by the union of CTVs on the corresponding 10-phase images of a 4D CT [33], with the volume varying from 22.8–194 cm^3^ with a median value of 54.5 cm^3^. For 34 Gy/fx plans, both the transmission and Bragg peak plans were generated using the same 5-beam arrangement with 72-degree equal intervals to give a uniform dose distribution to the target. For 18 Gy/fx plans, the beams were reduced to 4 with the identical angular arrangement between the transmission and Bragg peak plans. Four beams in the 54 Gy/3fx plans were used to achieve greater dose rates in each beam while still maintaining optimal plan quality and comparable target uniformity to plan with 5 beam angles. We first adopted a similar beam arrangement between transmission and Bragg peak plans (Figure 1a,b). Nevertheless, using a well-separated beam methodology is more favorable in transmission planning considerations, as closer or overlapping transmission beams may introduce a higher distal dose beyond the target, which could be avoided in the Bragg peak plans [23]. Moreover, in practice, shooting beams from or to the contralateral lung can be avoided in the Bragg peak plans, which may be challenging in transmission cases. As a result, we also compared differences in plan quality associated with beam angular arrangement (Figure 1c) within the Bragg peak plans.

### 2.3. Plan Quality Evaluation

#### 2.3.1. Dosimetry Analysis

The target coverage was normalized to 100% iCTV receiving at least 95% prescribed dose for comparison purposes. The RTOG 0915 [32] dose metrics were adopted to evaluate dosimetry parameters for the iCTV and OARs, including esophagus, spinal cord, heart, and lung-GTV (iCTV: D_0.2cc_; esophagus: D_5cc_, D_max_; spinal cord: D_0.35cc_, D_1.2cc_, D_max_; heart: D_15cc_, D_max_; lung-GTV: V_7Gy_, V_7.4Gy_).

#### 2.3.2. Dose Rate Quantification

The 3D dose rates of each beam in the treatment plans were computed based on DADR, ADR, and DTDR methods. Dose-rate volume histograms (DRVH) for CTV and each OAR were then calculated to evaluate the FLASH dose rate (>40 Gy/s) coverage. When using multiple fields to deliver FLASH treatment plans, the time spent between beams is much longer than each field’s dose delivery time. Therefore, the voxels having non-zero doses of each field in a plan are included for calculating the DRVH. We introduce the metric of V_40Gy/s_, i.e., the percent volume covered by FLASH dose rate (>40 Gy/s), as the variable of merit.

##### Dose-Averaged Dose Rate (DADR)

Van der Water et al. [18] first proposed the dose-averaged dose rate (DADR) method in head and neck patients. The DADR is defined in Equation (1), adopted for dose rate quantification:

(1) $${\dot{D}}_{j}^{DADR}=\overset{N}{\underset{i=1}{\sum }}\;\;\frac{D_{j,i}}{\sum_{i=1}^{N}D_{j.i}}{\dot{D}}_{j.i}$$

Here, *i* denotes a spot, j denotes a voxelized region in the target and D*_j.i_* is the dose deposited by the *i*-th spot to the *j*-th voxel.

(2) $${\dot{D}}_{j.i}={\dot{D}}_{max}e^{-\frac{{\left(r_{j}-r_{i}^{c}\right)}^{2}}{\sigma^{2}}}$$

${\dot{D}}_{max}$ is the dose rate at the spot central axis that scales the following Gaussian dose fall-off in the spot lateral direction at a particular depth, as shown in Equation (2), where $r_{i}^{c}\;$is the position of i-th spot center, $r_{j}$ is the position of the *j*-th voxel, σ is the spot sigma. Note that the ${\dot{D}}_{max}$ is a varying factor with respect to the radiological path in a 3D volume, which can be determined by a dose calculation engine.

##### Average Dose Rate (ADR)

The average dose rate idea was first proposed by Folkerts et al. [19], where the dose rate is averaged over the period for scanning the field. The dose rate calculation formula is shown in Equation (3) for a particular voxel *j*,$\;D_{j}$ is the total dose deposited in voxel *j* during the irradiation, $d^{*}\;$is a preset dose threshold that determines the irradiation start time corresponding to the low dose threshold $d^{*}\;$and the end time corresponding to the high dose threshold $D_{j}-d^{*}$. The $d^{*}\;$was chosen as 0.1 Gy by Folkerts et al. [19] in their work.

(3) $${\dot{D}}_{j}^{ADR}=\frac{D_{j}-2d^{*}}{T_{j}}$$

where,

$$d_{j}\left(t_{0}\right)=d^{*}\;d_{j}\left(t_{1}\right)=D_{j}-d^{*}\;T_{j}=t_{1}-t_{0}$$

Therefore, the average dose rate is the quotient between the accumulated dose and the period within the low and high dose thresholds.

##### Dose Threshold Dose Rate (DTDR)

The FLASH sparing effect has previously been suggested to be related to dose rate and may be combined with dose threshold [27,28,29]. Wilson et al. [27] have shown that for oxygen concentrations of 0.4%, the dose of 5–10 Gy is sufficient to deplete cellular oxygen at the FLASH dose rate. In [29], the in vitro evidence shows that for a particular hypoxic condition (1.6% oxygen concentration), the FLASH-sparing effect starts at 5–10 Gy, is apparent at ≥15 Gy, and is significant at 18 Gy. The result suggests no survival fraction difference for doses <5 Gy between FLASH RT and conventional RT. PBS delivery might be relevant to use a dose-threshold to exclude the low dose tails of the PBS spots that deposit less dose than a predefined dose-threshold from the instantaneous dose rate calculation for a region of interest (i.e., a voxel). Based on the above observations and postulation, Kang et al. [20] proposed the dose threshold dose rate (DTDR), as shown in Equation (4). For an arbitrary voxel *j*, the dose-threshold dose rate (DTDR) is the minimum instantaneous dose rate of all the spots that deposit the dose to the voxel above a predefined dose threshold. In this work, we assumed 0.1 Gy for the dose threshold $d^{*}$.

(4) $${\dot{D}}_{j}^{DTDR}=\min\left({\dot{D}}_{j,i}\right),\;\mathrm{if}\;D_{j,i}>d^{*},\;i=1,2\ldots n$$

We conservatively assumed the FLASH effect was only achieved in a field location when the dose rates from all spots delivering the above dose threshold are above the 40 Gy/s dose rate threshold.

**Figure 2:**
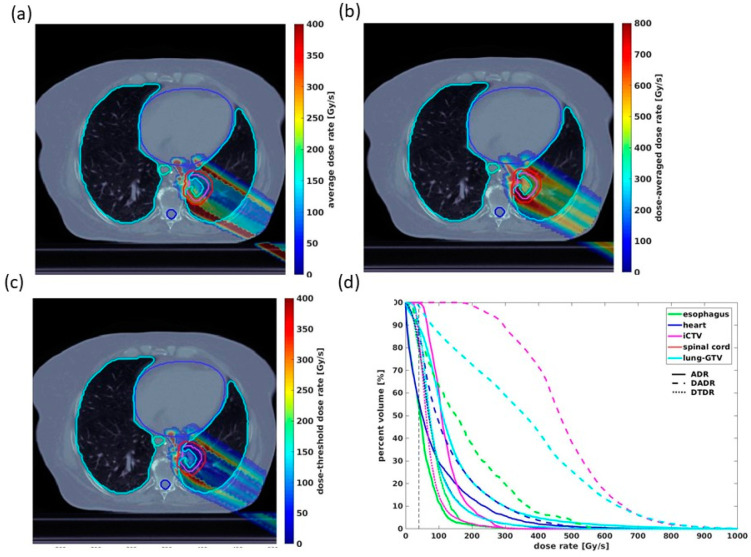
An axial slice of one field of the 3D dose rate distribution of one Bragg-peak plan corresponding to (**a**) ADR, (**b**) DADR, and (**c**) DTDR calculations. (**d**) DRVHs of the three-dose-rate, indicating differences in the dose rate distributions, with intersections of the 40 Gy/s dose rate threshold plotted by the dashed line.

The overall implementation of the above dose rate calculations and DRVH method is indicated in Figure 2 as a representative example. Figure 2a–c demonstrates one field of the dose rate map following the three definitions superimposed on the same CT slice. The different definitions evidently give distinct quantitative results in dose rate coverage, with DADR achieving the highest dose rates in most voxels, followed by DTDR and ADR. Figure 2d demonstrates the corresponding DRVHs for the same field of three-dose-rate distributions, indicating consistent observations with the dose rate maps. For multiple fields, as in our lung plans, the dose rates and voxels associated with each field are combined into one larger container for DRVH calculations of each contour, from which the V_40Gy/s_ can be further obtained.

## 3. Results

### 3.1. Dosimetry Analysis

**Figure 3:**
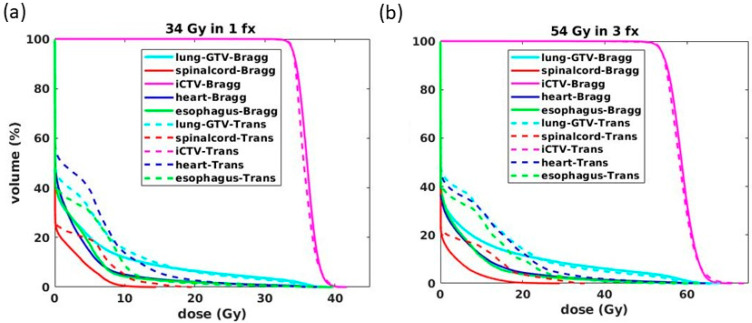
Averaged DVHs of (**a**) 34 Gy/fx and (**b**) 18 Gy/fx Bragg peak (solid lines) and transmission (dashed lines) plans.

For the minimum MU/spot of 400 and 1200, the transmission and Bragg peak plans yielded similar CTV uniformity indicated with D_0.2cc_. For 34 Gy/fx, the D_0.2cc_ is 112.9 ± 2.6% for Bragg peak plans and 111.5 ± 3.8% for the transmission plans. For 18 Gy/fx, the D_0.2cc_ is 119.4 ± 2.8% for the Bragg peak plans and 118.1 ± 5% for the transmission plans, also indicated in Figure 3a,b. In general, CTV uniformity was superior in 34 Gy/fx compared to the 18 Gy/fx plans, as the 34 Gy/fx plans employed 5 fields and delivered higher doses in each field, which allows more flexibility in spot weight optimization. As shown in Figure 3, in both 34 Gy/fx and 18 Gy/fx cases, the Bragg peak plans achieved a much lower dose volume for all OARs compared with transmission plans, especially for the low (0–5 Gy) to medium (5–15 Gy) dose regions.

**Figure 4:**
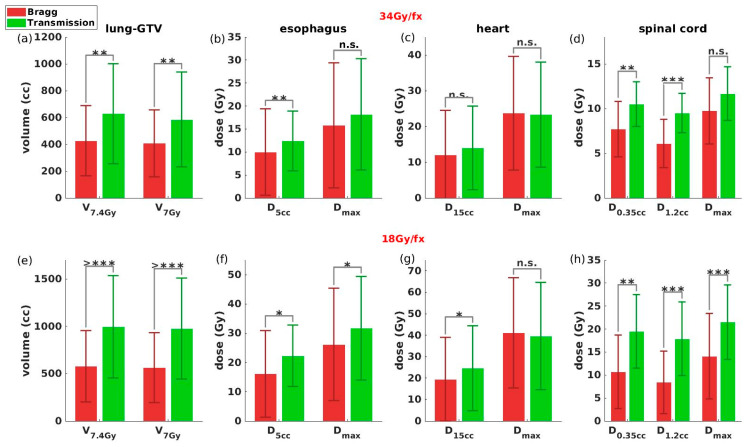
Barplots of dosimetry parameters for lung-GTV, esophagus, heart, and spinal cord in the 34G y/fx ((**a**)–(**d**)) and 18 Gy/fx ((**e**)–(**h**)) Bragg peak and transmission plans. * indicates *p* < 0.05, ** indicates *p* < 0.01, *** indicates *p* < 0.001, >*** indicates *p* < 0.0001, and n.s. indicates non-significant.

Statistical results are shown in Figure 4. The most significant dose reduction was observed in the lung-GTV. The Bragg peak plans achieved 32.0% (*p* < 0.01) and 30.4% (*p* < 0.01) reductions in V_7.4Gy_ and V_7Gy_ in 34 Gy/fx, respectively, and 40.8% (*p* < 0.01) and 41.2% (*p* < 0.01) in 18 Gy/fx, respectively. For the spinal cord, reductions of 26.6% (*p* < 0.01), 35.9% (*p* < 0.01) and 16.6% were observed in D_0.35cc_, D_1.2cc_, and D_max_ in 34 Gy/fx, respectively, and 45.0% (*p* < 0.01), 52.8% (*p* < 0.01), and 34.4% (*p* < 0.01) in 18 Gy/fx, respectively. The Bragg peak plans also generated significantly better D_5cc_ in the esophagus and a slightly better average D_15cc_ for the heart. The slightly lower D_max_ in transmission plans relative to Bragg peak plans was not statistically significant.

### 3.2. Dose Rate Quantification

As shown in the bottom line of Table 1, the average V_40Gy/s_ is lowest using the ADR method compared to DADR and DTDR for all OARs in both the Bragg peak and transmission plans and both fractionations. In contrast, the DADR method achieves the highest average V_40Gy/s_ in the Bragg peak cases and is comparable to the DTDR in the transmission cases.

Overall, the average V_40Gy/s_ differences for average OARs between the Bragg peak and transmission methods in DADR was ~3.7% in 34 Gy/fx cases and ~2.7% in the 18 Gy/fx plans. The differences became more evident in the DTDR (~13.4% for average OARs) and ADR (~8.8% for average OARs) for the 34 Gy/fx plans and were ~7.4% in the DTDR and 14.7% in the ADR for the 18Gy/fx plans. 

**Figure 5:**
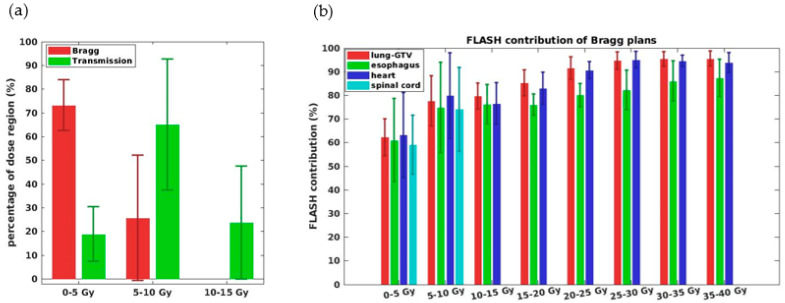
(**a**) Ratio of spinal cord volume in different dose regions for both Bragg peak and transmission plans, noting that the spinal cord has a larger portion receiving dose between 0–5 Gy in the Bragg peak plans than the transmission plans. (**b**) FLASH dose rate coverage V40 Gy/s (based on ADR calculations) distribution vs. different dose regions for major OARs. Note the low dose region of the OARs 0–5 Gy is associated with the lowest FLASH contributions.

The relatively large differences (such as 16.8% ADR differences) for the spinal cord in 34 Gy/fx can be explained by the discrepancies between the Bragg peak and transmission plans of low-dose volume coverage (Figure 5a), due to the sparing of OARs beyond the target in the Bragg peak plans and differential FLASH contributions to dose levels (Figure 5b). As shown in Figure 5b, we note that FLASH contribution increases as dose increases in the Bragg peak plans. We also note in Figure 5a that there is much more 0–5 Gy portion (~70% in Bragg peak vs. ~20% in transmission, excluding 0 Gy in the OARs) associated with the lowest ADR in the Bragg peak plans than in the transmission plans.

### 3.3. Beam Angular Optimization in the Bragg Peak Planning

The CTV D_0.2cc_ of the optimized beam angular Bragg peak plans was 117.6 ± 2.8% compared to 119.3 ± 2.8% for the unoptimized plans. There was a trend towards improving target uniformity with beam angular optimization, yet it did not achieve statistical significance.

**Figure 6:**
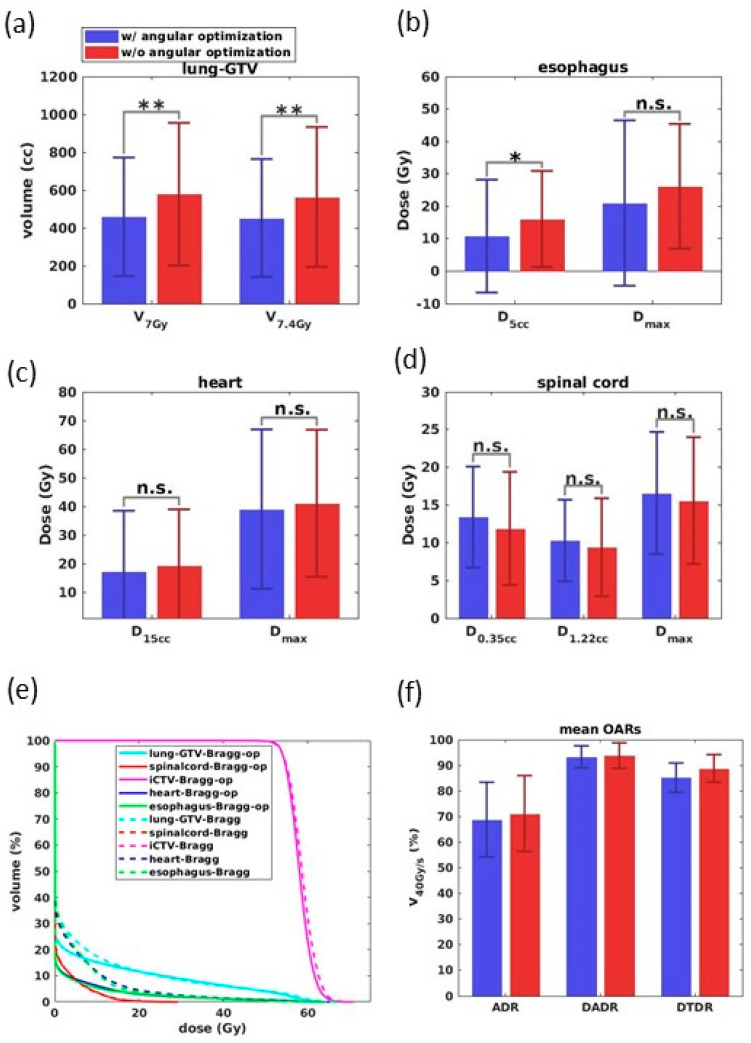
Barplots of dosimetry parameters for (**a**) lung-GTV, (**b**) esophagus, (**c**) heart, and (**d**) spinal cord in the Bragg peak plans with (blue) and without (red) beam angular optimization. Here, * indicates *p* < 0.05, ** indicates *p* < 0.01, and n.s. indicates non-significant. (**e**) With (solid line) and without (dashed line) angular optimization comparison by averaging the DVHs of all ten patients’ Bragg peak plans. (**f**) Barplot of the mean V 40 Gy/s for all OARs based on the three-dose-rate results.

As shown in Figure 6a–d, with optimized beam angular arrangements, the Bragg peak plans achieve, on average, 20.7% (*p* < 0.01) and 19.7% (*p* < 0.01) reductions in the lung V_7Gy(cc)_ and V_7.4Gy(cc)_, respectively. The dosimetry parameters for other OARs were improved as well with beam angular optimization. Figure 6e shows the averaged DVHs of the Bragg peak plans with and without angular optimization. We can identify the Bragg peak plans’ superior dosimetry performances with angular optimization in the low to medium dose range for the lung, heart, and esophagus.

For the dose rate results (Figure 6f) that we found in most OARs, the V_40Gy/s_ associated with beam angular optimization are comparable to those without optimization for all dose rate calculations, and the dose rate differences are less than 5%.

## 4. Discussion

This study systematically evaluated the dosimetry performances and dose rate results between Bragg peak and transmission plans for ten hypofractionated lung cancer patients using proton SBRT. The planning tool we developed allows us to optimize the Bragg peak and transmission plans to achieve acceptable plan quality and FLASH dose rates. The Bragg peak plans’ advantages in sparing the tissue beyond the target in the beam path are comprehensively demonstrated in comparison with the transmission plans. The Bragg peak plans are especially beneficial for lung cancer treatment by reducing the low to middle dose range to the normal lung tissues.

We adopted the same angular arrangement and beam numbers for all Bragg peak and transmission plans to minimize potential bias when comparing the dosimetry between plans. In doing so, this study shows that using 4–5 beams for transmission plans is sufficient to generate acceptable plan quality and adequate FLASH RT dose rates. Given the sizes and locations of lung tumors, this study also identifies that different beam angular arrangements in transmission plans do not significantly affect lung dosimetry parameters and target coverage. While nearly all pre-clinical FLASH data to date has employed only a single fraction [34], this study also indicates that using a smaller dose fraction, such as 18 Gy/fx, requires more consideration in designing the optimal number of fields, spot spacing, and optimization procedures (such as spot merging) to achieve sufficient dose rate and acceptable plan quality when using Bragg peak planning. Future investigation will assess the impact of fractionation on dose rate and plan quality for additional disease sites.

Different dose rate quantification methods were evaluated in this work. We observe different outcomes across the dose rate methods but similar trends between Bragg peak and transmission plans. Our study provides a comprehensive evaluation of the FLASH dose rates because unique but essential factors are accounted for in PBS, including spot scanning sequences (zigzag scanning patterns), beam-on time, spot switching effect, etc., which could provide useful information to correlate with biological endpoint results. We also identified that FLASH dose rates do not always cover the entire OARs in both transmission and Bragg peak methods. As indicated in the results, this could be due, in large part, to the relatively lower dose rates associated with lower dose regions where it is expected that there will be no FLASH RT sparing effect [27,28,29]. It is, however, possible to optimize the minimum MUs/spot to increase the FLASH dose rate coverage in OARs as much as possible while retaining similar plan quality. For ADR in particular, it is also possible to optimize the scanning patterns to boost the dose rate coverage in OARs. Such dose rate optimizations require separate and further study in the future. The current novel study, however, has shown that Bragg peak methods with a minimum 1200 MU/spot could achieve comparable FLASH dose rate coverage in OARs compared to transmission plans while also achieving comparable iCTV coverage and superior OAR dosimetry performances with the specific planning optimization procedures that we developed. DTDR is a new concept assessed in this work. For simplicity, we assumed a 0.1 Gy threshold in this study. In reality, this value could be tissue-specific and may need additional information, such as dwelling time between spots, etc., which is not studied here.

Currently, Bragg peak plans require a higher minimum MU/spot, i.e., nozzle current, compared to the transmission plans, which may not be readily achievable with most proton machines. Other FLASH-relevant parameters (such as spot switch time) need to be verified with the current proton systems. New designs or hardware upgrades (to improve the beam transportation efficiency or boost the beam current) will be critical to enable this novel FLASH delivery method for future clinical applications [35]. The Bragg peak method uses the highest energy beams of the cyclotron. The energy straggling due to stochastic energy loss from inelastic Coulomb interactions between protons and URS/RC/tissue produces a broader energy spectrum [30]. As there is no energy selection to eliminate protons to narrow the proton beams in the energy spread, the Bragg peak width becomes wider. The highest energy of 250 MeV proton beams travels about 380 mm in water (water equivalent tissue), resulting in constant peak width and distal dose fall-off for all treatment fields.

It is also critical to be aware of the real challenges in implementing Bragg peak treatment plans, especially range uncertainties associated with range shifter design, precise positioning of the patient, CT calibrations, HU to stopping power conversion, etc. Motion management also plays an essential role in ensuring the plan’s robustness. The beam-on time of <1 s for each field grants breathhold during FLASH treatment, and as a result, the intrafraction motion may not be a significant concern, but the interfraction or inter-field target motion still needs to be considered. For instance, an average image from a 4DCT, which allows targets to be contoured over motion, may make the treatment plan more robust [33]. Other techniques, such as gating or deep inspiration breath-hold (DIBH) [36], may reduce the target margin and interplay effect to achieve a more robust treatment [37].

Currently, Bragg peak and transmission FLASH proton plans have adopted multiple fields to deliver the prescribed dose in one or multiple fractions, whereas in all of the pioneering pre-clinical or human FLASH experiments [1,2,3,4,5,6], only a single field and single fraction were used. How multiple fields or fractionations affect FLASH remains unclear [18], which needs to be assessed by collaborations in the community to advance FLASH RT into clinical practice safely and effectively.

## 5. Conclusions

Single-energy pencil beam scanning proton Bragg peak FLASH plans can achieve superior OAR plan quality compared with transmission plans for hypofractionated lung cancer cases due to its sparing of normal tissue distal to the target in the beam path. This novel FLASH treatment method also retains an adequate FLASH dose rate compared to the transmission plans, enabled by a boosted yet feasible minimum MU/spot, i.e., nozzle current, in a field, along with dedicated inverse optimization planning featuring a sophisticated and iterative dose rate optimization process. The Bragg peak plans achieve superior dosimetry and similar dose rate performance despite the differences in dose fractionations, and even further improvement of Bragg peak plan quality can be achieved with optimized beam angular arrangement, which allows for more optimal sparing of normal tissues and may be an attractive planning approach for future human clinical trials.

## Figures and Tables

**Table 1:** V_40Gy/s_ results using different dose rate calculations for both the Bragg peak and transmission plans for 34 Gy/fx and 18 Gy/fx fractionations.

	34 Gy/fx						18 Gy/fx					
	Bragg Peak			Transmission			Bragg Peak			Transmission		
	ADR	DADR	DTDR	ADR	DADR	DTDR	ADR	DADR	DTDR	ADR	DADR	DTDR
Lung-GTV	81.0 ± 4.6	96.7 ± 0.8	86.6 ± 3.9	84.7 ± 4.6	97.7 ± 0.7	97.6 ± 0.5	81.0 ± 2.9	97.4 ± 0.8	89.9 ± 2.9	86.6 ± 4.7	98.1 ± 0.5	98.0 ± 0.4
Esophagus	74.5 ± 9.3	94.8 ± 2.4	84.1 ± 5.0	76.5 ± 9.8	96.7 ± 4.4	96.3 ± 2.4	71.5 ± 17.1	95.1 ± 3.4	92.1 ± 4.3	81.3 ± 11.1	98.5 ± 0.8	97.5 ± 1.2
Heart	76.7 ± 9.5	90.4 ± 13.6	84.7 ± 13.5	82.1 ± 12.2	96.6 ± 3.7	96.8 ± 2.0	70.8 ± 12.5	86.7 ± 5.1	90.2 ± 4.7	86.7 ± 5.1	96.3 ± 4.5	95.9 ± 3.3
Spinal cord	62.8 ± 15.9	90.9 ± 4.6	79.5 ± 8.6	79.6 ± 7.5	96.5 ± 2.1	96.1 ± 2.3	62.2 ± 18.2	93.4 ± 4.2	86.1 ± 6.9	83.0 ± 6.3	98.4 ± 1.3	95.8 ± 2.9
Average OARs	73.6 ± 12.4	93.3 ± 7.3	83.7 ± 8.5	80.7 ± 9.0	96.9 ± 3.0	96.7 ± 2.0	71.9 ± 14.8	95.3 ± 3.3	89.6 ± 5.1	84.3 ± 7.4	97.9 ± 2.4	96.8 ± 2.4

## Data Availability

The data presented in this study are available on request from the corresponding author.
